# Adenocarcinoma originating in the anterior mediastinum diagnosed by endobronchial ultrasound-guided transbronchial cryobiopsy: a case report

**DOI:** 10.1186/s12890-024-02998-8

**Published:** 2024-04-16

**Authors:** Hiroyuki Tsuchida, Yuji Matsumoto, Hideaki Furuse, Takaaki Tsuchida

**Affiliations:** 1 Department of Endoscopy, Respiratory Endoscopy Division, National Cancer Center Hospital, 5-1-1 Tsukiji, Chuo-Ku, Tokyo, Japan; 2 Department of Thoracic Oncology, National Cancer Center Hospital, 5-1-1 Tsukiji, Chuo-Ku, Tokyo, Japan

**Keywords:** Bronchoscopy, Cryobiopsy, Endobronchial ultrasound-guided transbronchial needle aspiration, Computed tomography-guided needle biopsy, Anterior mediastinal tumor

## Abstract

**Background:**

Endobronchial ultrasound-guided transbronchial cryobiopsy (EBUS-cryobiopsy) is advantageous for collecting larger specimens with minimal crushing; however, it has not been widely used for mediastinal tumors.

**Case presentation:**

A 73-year-old woman with a history of left breast cancer underwent surgery followed by radiotherapy. Computed tomography showed a mass in the anterior mediastinum that was in extensive contact with the sternum on the ventral side and partly with the trachea on the dorsal side. Two computed tomography-guided needle biopsies (CTNBs) were performed on the mass; however, a definitive diagnosis was not made because of severe crush artifacts. Subsequently, we performed EBUS-cryobiopsy and safely obtained sufficient specimen volume with minimal crushing. The histopathological diagnosis was adenocarcinoma, with immunobiological features distinct from those of previous breast cancers. Her overall diagnosis was a rare tumor originating in the anterior mediastinum.

**Conclusions:**

EBUS-cryobiopsy can be safely performed in narrow areas surrounded by major blood vessels, and the obtained specimens may be superior to CTNBs for histopathological diagnosis.

## Background

Computed tomography-guided needle biopsy (CTNB) is the first-choice diagnostic method for mediastinal lesions that can be approached from the chest wall [1]. Endobronchial ultrasound-guided transbronchial needle aspiration (EBUS-TBNA) is a standard sampling technique for mediastinal and hilar lesions. However, because only cytological specimens are obtained with an aspiration needle, the diagnostic yield is limited. Recently, EBUS-guided transbronchial cryobiopsy (EBUS-cryobiopsy) has been reported to overcome these limitations.

We report a case of a patient with adenocarcinoma that originated in the anterior mediastinum adjacent to the trifurcation of the aortic arch and was diagnosed by cryobiopsy.

## Case presentation

### Patient information

**Fig. 1:**
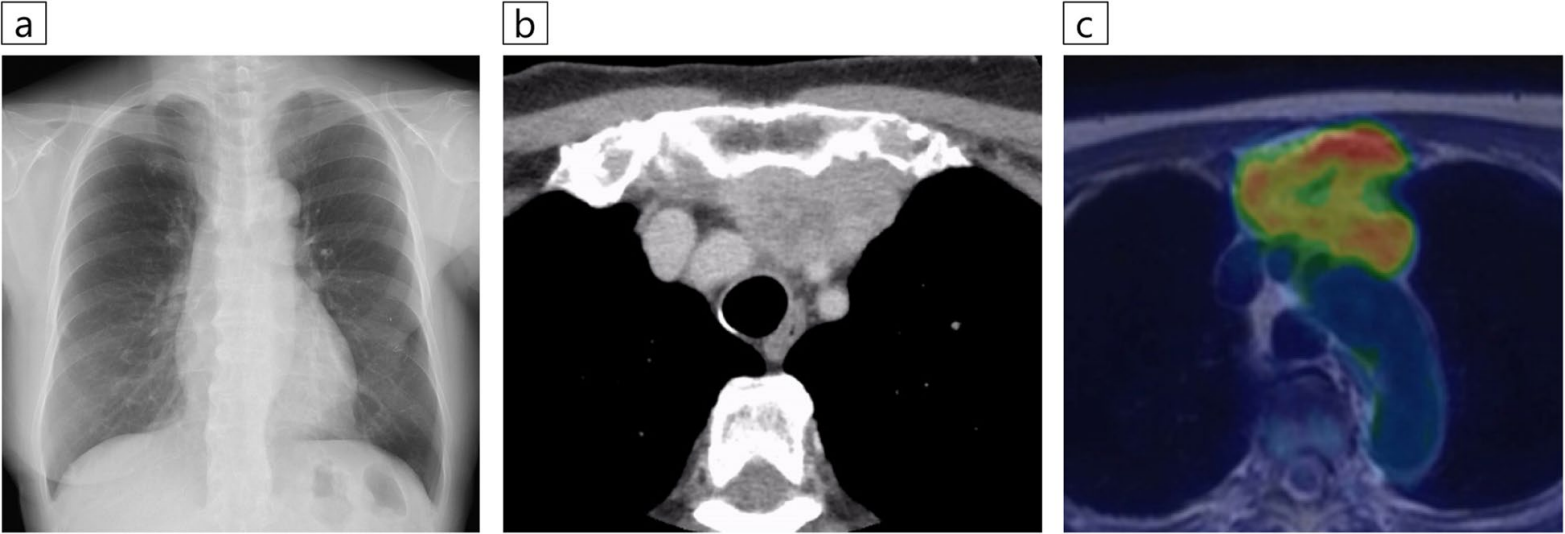
Radiographic imaging findings. **a** Chest X-ray showed an abnormal opacity in the mediastinum at the patient’s first visit to our hospital. **b** Chest contrast-enhanced computed tomography showed a mass with a maximum diameter of 5.2 cm in the anterior mediastinum. The lesion was extensively in contact with the sternum on the ventral side and partly in contact with the trachea on the dorsal side, between the brachiocephalic and the left common carotid arteries. **c **^18^F-fluorodeoxyglucose-positron emission tomography (FDG-PET) showed high FDG uptake (maximum standardized uptake value was 6.71) in the anterior mediastinal mass and sternal infiltrate

The patient was a 73-year-old woman, who had never smoked, and who had a history of left breast cancer that was treated with surgery followed by radiotherapy. Chest radiography revealed an abnormal shadow in the mediastinum (Fig. 1a). There were no notable physical findings or abnormal values of various tumor markers.

### Clinical findings

**Fig. 2:**
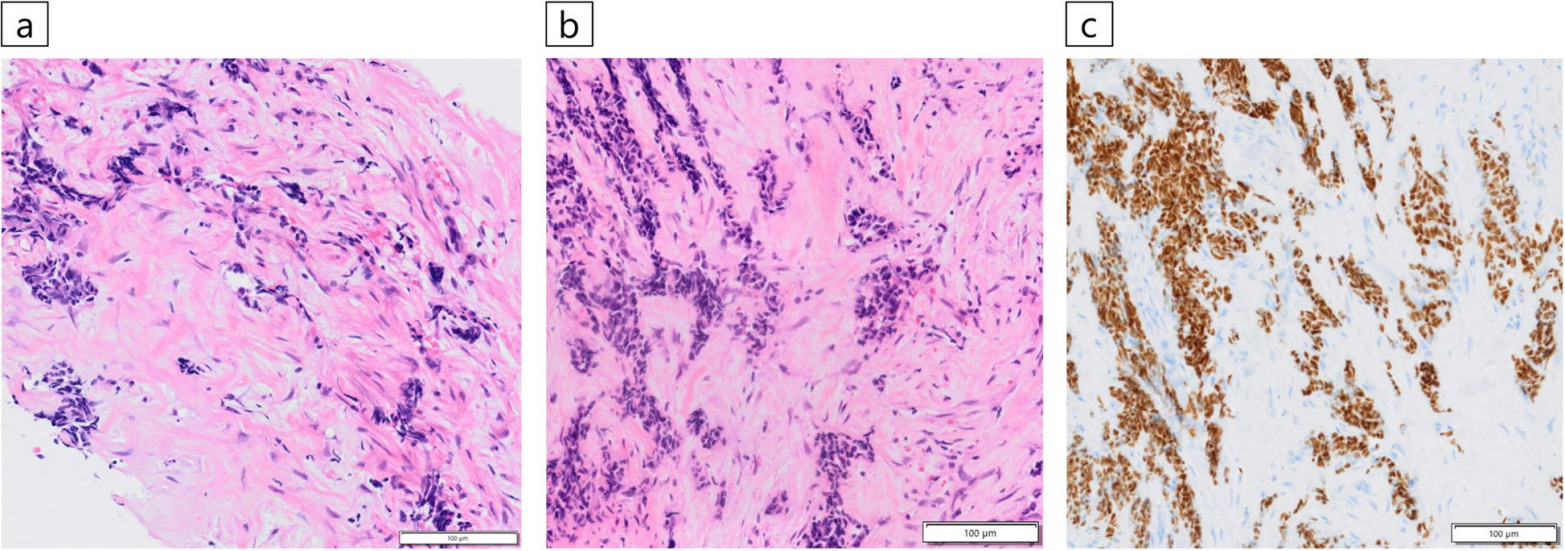
Histopathological findings. **a** An analysis of computed tomography-guided needle biopsy specimens did not lead to a definitive diagnosis due to severe tissue crushing (Hematoxylin and eosin staining). **b** The endobronchial ultrasound-guided transbronchial cryobiopsy specimens presented minimal crushing, and proliferating tumor cells with chromatin-enriched micronuclei were identified that formed scattered small foci against a background of extensive fibrous connective tissue with hyaline changes (Hematoxylin and eosin staining). **c** Immunohistochemical staining was positive for HNF4α, which is a finding suggestive of adenocarcinoma of gastrointestinal origin

Chest contrast-enhanced computed tomography (CT) performed prior to ablation therapy for atrial fibrillation showed a mass with a maximum diameter of 5.2 cm in the anterior mediastinum. The mass was in extensive contact with the sternum on the ventral side and partly with the trachea on the dorsal side between the brachiocephalic and left common carotid arteries (Fig. 1b). ^18^F-fluorodeoxyglucose-positron emission tomography (FDG-PET) showed high FDG uptake in the mass and sternal infiltration but no significant uptake elsewhere (Fig. 1c). Two CTNBs were performed on the mass, but a definitive diagnosis was not made because of severe crush artifacts (Fig. 2a). We then performed EBUS-cryobiopsy to diagnose the lesion.

### Diagnostic assessment

**Fig. 3:**
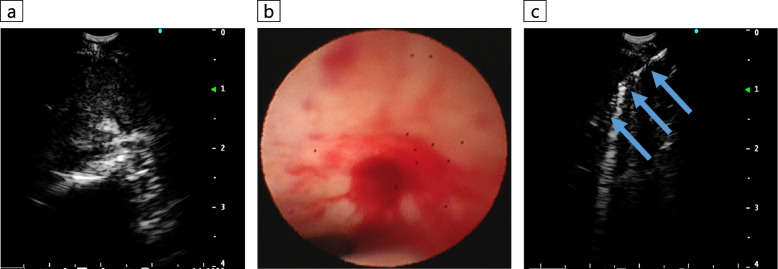
Endobronchial ultrasound-guided transbronchial cryobiopsy (EBUS-cryobiopsy) procedure images. **a** The anterior mediastinal mass was visualized by EBUS with reference to the locations of the brachiocephalic and left common carotid arteries. **b** A small tract was made in the tracheal wall while sampling with EBUS-guided transbronchial needle aspiration using a 22-gauge needle. **c** EBUS-cryobiopsy was performed by inserting a cryoprobe with a diameter of 1.7 mm (arrow) into the lesion through the created tract

Using a linear EBUS bronchoscope (BF-UC290F, Olympus, Japan), the lesion was delineated from the trachea while observing the brachiocephalic and left common carotid arteries as merkmals, and a 22-gauge needle (NA-U401SX, Olympus, Japan) was used to create a small tract in the tracheal wall while collecting specimens with EBUS-TBNA [2]. Subsequently, EBUS-cryobiopsy was performed four times with freezing for around five seconds each time by inserting a 1.7-mm cryoprobe (20402–410, Erbe Elektromedizin GmbH, Germany) into the lesion through the tract, which was completed without complications (Fig. 3). EBUS-cryobiopsy yielded sufficient specimen volume with minimal crushing. Histopathological findings revealed proliferating tumor cells with chromatin-enriched micronuclei that formed scattered small foci against a background of extensive fibrous connective tissue with hyaline changes, indicating adenocarcinoma (Fig. 2b). The immunohistochemical staining features were distinct from those of the past breast cancer and thymic epithelial tumors, although HNF4α, CDX2, and CK20 were positive, which suggested a gastrointestinal tumor (Fig. 2c). However, it was difficult to morphologically determine whether the metastasis originated from a gastrointestinal tumor, and the systemic examination was negative for the presence of a gastrointestinal tumor. The tumor was diagnosed as a rare adenocarcinoma originating in the anterior mediastinum.

### Therapeutic intervention

The patient underwent chemotherapy without any late complications of EBUS-cryobiopsy.

## Discussion

We report a case of a patient with adenocarcinoma that originated in the anterior mediastinum and was diagnosed using EBUS-cryobiopsy. Epithelial tumors that occur in the anterior mediastinum include thymic carcinomas, metastases from other carcinomas, and primary anterior mediastinal carcinomas. In this patient, immunochemical staining was negative for PAX8, p40, ER, and PgR, ruling out thymic carcinoma and breast cancer metastasis. The immunohistochemistry suggested the adenocarcinoma was of gastrointestinal origin, which was ruled negative after thorough systemic examination. The tumor was diagnosed as an adenocarcinoma that originated in the anterior mediastinum. Mucinous adenocarcinomas associated with thymic cysts have been sporadically reported among primary anterior mediastinal adenocarcinomas; however, no prior reports have documented the correlation with HNF4α. To the best of our knowledge, this is the first case of a patient with adenocarcinoma that lacked thymic cysts and presented with HNF4α positivity.

CTNB has been reported to have a diagnostic yield of 94% for mediastinal tumors and should be considered as the highest priority when the tumor is in contact with or near the chest wall [1]. Previous reports have stated that using thinner needles (i.e., 20-gauge) and obtaining less than three samples were significant risk factors for CTNB diagnostic failure [3]. For this patient, four and six samples were collected using 18-gauge needles on the first and second attempts, respectively. Furthermore, diagnostic yields have been observed to be low in patients with malignant lymphoma [3]. We hypothesize that the diagnostic failure was due to extensive fibrous connective tissue within the lesion, which made the tumor highly susceptible to crushing. Also, it has been reported that mediastinoscopy biopsy has higher diagnostic accuracy than needle biopsy [4]. However, it is a fairly invasive procedure due to rib resection, incision location and length, longer follow-up time, and possible pleural opening [5]. Therefore, a less invasive transtracheal approach should be prioritized.

While EBUS has a high diagnostic yield due to real-time guidance, the yield by conventional TBNA is limited for some diseases. Recently, the efficacy of cryobiopsy, which allows for large-quantity and high-quality sampling, has been reported in two randomized controlled trials [6, 7]. Notably, we were able to diagnose the rare tumor mentioned above by performing EBUS-cryobiopsy. EBUS-cryobiopsy provides real-time results and can be performed safely, even for lesions that border major vessels, whereas conventional cryobiopsy for lung lesions carries the risk of bleeding.

## Conclusion

Our findings suggest that EBUS-cryobiopsy can be safely performed in narrow areas surrounded by major blood vessels, and that the obtained specimens may be superior to those from CTNB for histopathological diagnosis.

## Data Availability

All data generated or analyzed during this study are included in this article. Further enquiries can be directed to the corresponding author.

## Abbreviations

CT: Computed tomography
CTNB: Computed tomography-guided needle biopsy
EBUS-cryobiopsy: EBUS-guided transbronchial cryobiopsy
EBUS-TBNA: Endobronchial ultrasound-guided transbronchial needle aspiration
FDG-PET: 18F-fluorodeoxyglucose-positron emission tomography
